# Cryo-EM analysis of cooperative conformational changes in the SARS-CoV-2 spike protein trimer

**DOI:** 10.1107/S2052252526004513

**Published:** 2026-06-25

**Authors:** Qiyu Wang, Spencer Cholak, Geoffrey Woollard, Sriram Subramaniam, Khanh Dao Duc

**Affiliations:** a Department of Computer Science University of British Columbia Vancouver BC V6T 1Z3 Canada; b Department of Biochemistry and Molecular Biology University of British Columbia Vancouver BC V6T 1Z3 Canada; c Department of Mathematics University of British Columbia Vancouver BC V6T 1Z3 Canada; Max Planck Institute of Biophysics, Germany

**Keywords:** SARS-CoV-2 receptor-binding domain, cryo-electron microscopy, heterogeneity analysis, structural biology

## Abstract

The entry of SARS-CoV-2 into cells is mediated by a trimeric spike protein that utilizes its receptor-binding domain (RBD) to engage with the cell surface receptor ACE2, with each protomer capable of shuttling between an ‘up’ conformation that can bind ACE2 and a ‘down’ conformation that prevents ACE2 binding. To discover inter­mediate conformational states during this transition, we applied and refined a recently reported linear subspace method to embed 2D projection cryo-EM images of ACE2-bound spike proteins into a low-dimensional latent space. From this embedding, multiple conformational states were reconstructed followed by an automated masking protocol, leveraging a topology representing network and optimal transport. A surprising result from our analysis is the finding that the dominant trajectory for the transition between conformations that have one-up and two-up RBD states involves an all-down RBD conformational state. The methods we present here could be generally applicable to other dynamic protein assemblies to uncover novel inter­mediate conformational states.

## Abstract 2

Analysis of SARS-CoV-2 spike protein heterogeneity using cryo-EM data reveals cooperativity between receptor-binding domains.

## 1. Introduction

**Figure 1:**
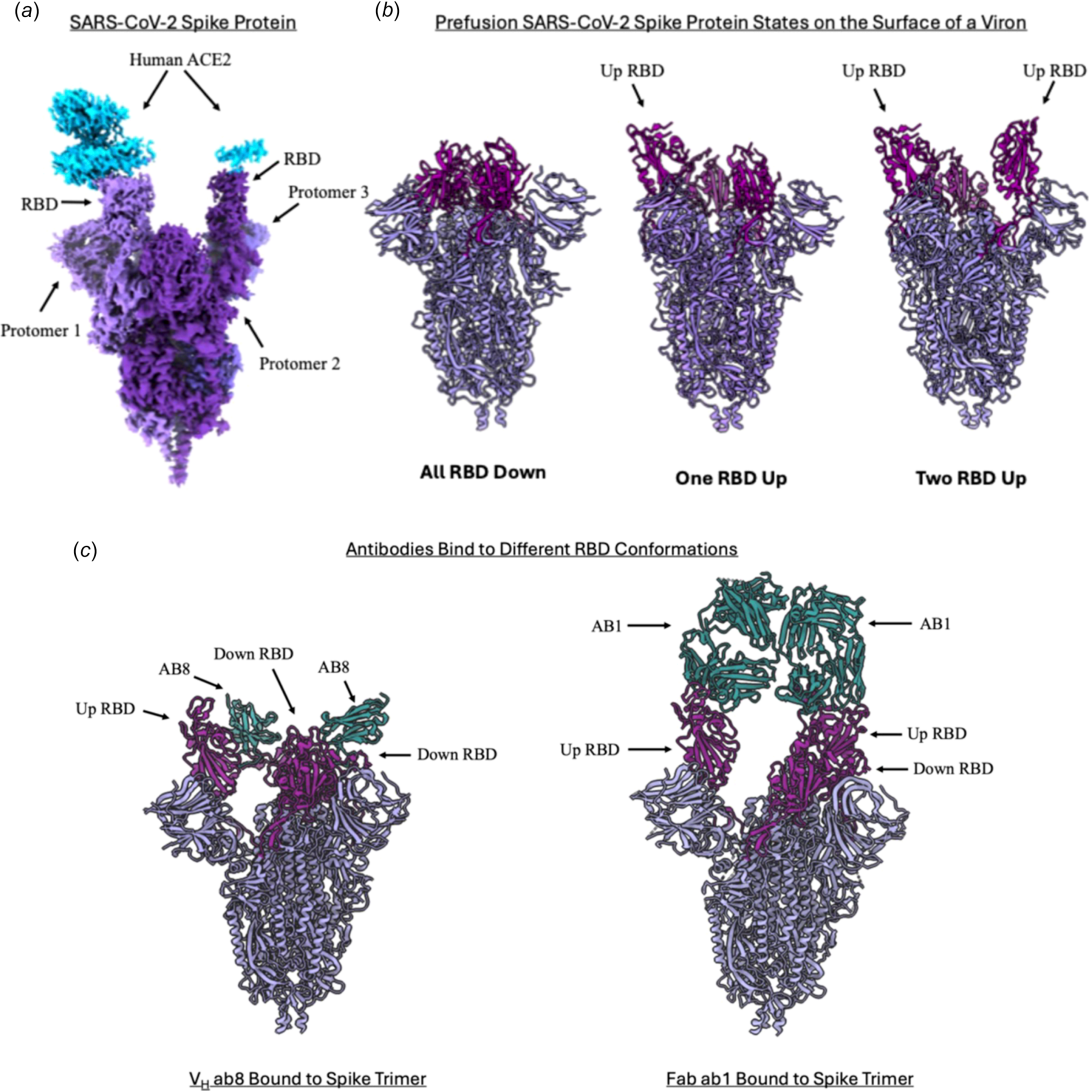
SARS-CoV-2 trimeric spike proteins are captured in discrete ‘RBD up’ and ‘RBD down’ states by single-particle cryo-EM and cryo-ET. (*a*) High-resolution structures of SARS-CoV-2 spike proteins in complex with ACE2 indicate a trimeric spike protein with ACE2 bound to up RBDs (EMDB-25760). (*b*) Cryo-ET of SARS-CoV-2 virions indicate that prefusion spike proteins can have all RBDs down, one RBD up, or two RBDs up in the absence of ACE2 (PDB IDs 6vyb, 6vxx and 6x2b) (Henderson *et al.*, 2020 ▸; Ke *et al.*, 2020 ▸; Walls *et al.*, 2020 ▸). (*c*) Atomic models of different RBD-directed neutralizing anti­bodies which have preferential binding to RBDs in the down and/or up position (PDB IDs 7mjh and 7mjj) (Zhu *et al.*, 2021 ▸).

The COVID-19 pandemic, caused by severe acute respiratory syndrome coronavirus 2 (SARS-CoV-2), has led to more than 7 million deaths up to May 2025 (Wu *et al.*, 2020 ▸; Cascella *et al.*, 2025 ▸; World Health Organization, 2025 ▸). As the pandemic spread, many efforts were dedicated to resolving the structure of the SARS-CoV-2 spike protein [S protein; Fig. 1 ▸(*a*)], which plays a crucial role in infecting human cells (Duan *et al.*, 2020 ▸; Huang *et al.*, 2020 ▸; Cerutti *et al.*, 2022 ▸; Zhu *et al.*, 2021 ▸; Mannar *et al.*, 2022 ▸; Saville *et al.*, 2022 ▸; Mannar *et al.*, 2024 ▸). The receptor-binding domain (RBD), present in each of the three protomers of the S protein, mediates binding to angiotensin-converting enzyme 2 (ACE2), a receptor widely expressed on the membranes of cells in the heart, testis, lung, nasal and oral mucosa­, nasopharynx, and muscle (Benton *et al.*, 2020 ▸; Lan *et al.*, 2020 ▸; Salamanna *et al.*, 2020 ▸).

### 1.1. Nomenclature of ‘up’ and ‘down’ states

The RBDs on each spike trimer can switch between ‘up’ and ‘down’ conformations [Fig. 1 ▸(*b*)], with the transition from down to up states also referred to sometimes as the ‘opening’ of the protein. The number of RBDs in the up and down conformation is described as the ‘RBD state’ of the spike protein. In the down conformation, the ACE2 binding surface of the S protein is blocked. In the open conformation, the S protein gains the ability to inter­act with ACE2 and facilitate cell entry. RBDs can act as potential targets of anti­body therapy (Barnes *et al.*, 2020 ▸; Min & Sun, 2021 ▸; Yuan *et al.*, 2021 ▸; Chen *et al.*, 2023 ▸). RBD-directed anti­bodies neutralize viruses by binding to and occluding receptor engagement [Fig. 1 ▸(*c*)]. RBD-directed anti­body binding is sensitive to the RBD conformational state, with anti­bodies such as ab8 binding to the down conformation and anti­bodies such as ab1 binding to the up conformation (Zhu *et al.*, 2021 ▸). Previous studies on RBD conformations of SARS-CoV-2 S proteins sug­gest that the conformational dynamics of RBDs are associated with viral infectivity and anti­body evasion (Valério *et al.*, 2022 ▸; Tang *et al.*, 2025 ▸).

Although discrete conformations of RBDs have been well characterized, a description of the cooperative engagement among the three RBDs in the conformational continuum between the various states remains elusive. As mentioned above, the SARS-CoV-2 S protein has three RBDs per trimer, each capable of transitioning between up/ACE2 bound and closed/no ACE2 bound states. Therefore, a single S protein trimer can be accessible for binding through states with one-up, two-up, or three-up RBDs (Zhao *et al.*, 2022 ▸; Lee *et al.*, 2023 ▸), although three-open RBDs are not frequently observed in structural studies with intact virions (Ke *et al.*, 2020 ▸) or in cryo-EM structures of purified spike protein trimers.

To explore inter­mediate states in the transition between down and up states, we used a data set of ∼700000 projection images extracted from cryo-EM images of spike protein trimers mixed with an excess of ACE2 protein. Under these conditions, we expect that there is enough ACE2 to bind each spike trimer that has one or more protomers in the up state. In our analysis, which is aimed at discovering trajectories and not discrete 3D structures, we therefore equate ACE2-bound protomers with up-state protomers for ease of description and refer to the states solely as being either up or down for each RBD protomer.

To characterize the potential continuous conformational changes between one-up and two-up RBD states, we used a com­pu­ta­tional approach to uncover novel inter­mediate con­formational states. Upon projecting particles from the dataset into a latent space continuously mapping 3D volumes with regularized covariance estimation for cryo-EM heterogeneity analysis (*RECOVAR*; Gilles & Singer, 2025 ▸), we found a significant presence of particles in both one-up and two-up RBD states, allowing us to extract trajectories between these states. By tracking the displacement of the RBDs along representative trajectories, we find that, surprisingly, the dominant trajectory from the one-up to two-up RBD states involves transition *via* an all-down RBD state.

## 2. Results

### 2.1. Heterogeneity analysis of cryo-EM density maps reveals multiple spike conformations

**Figure 2:**
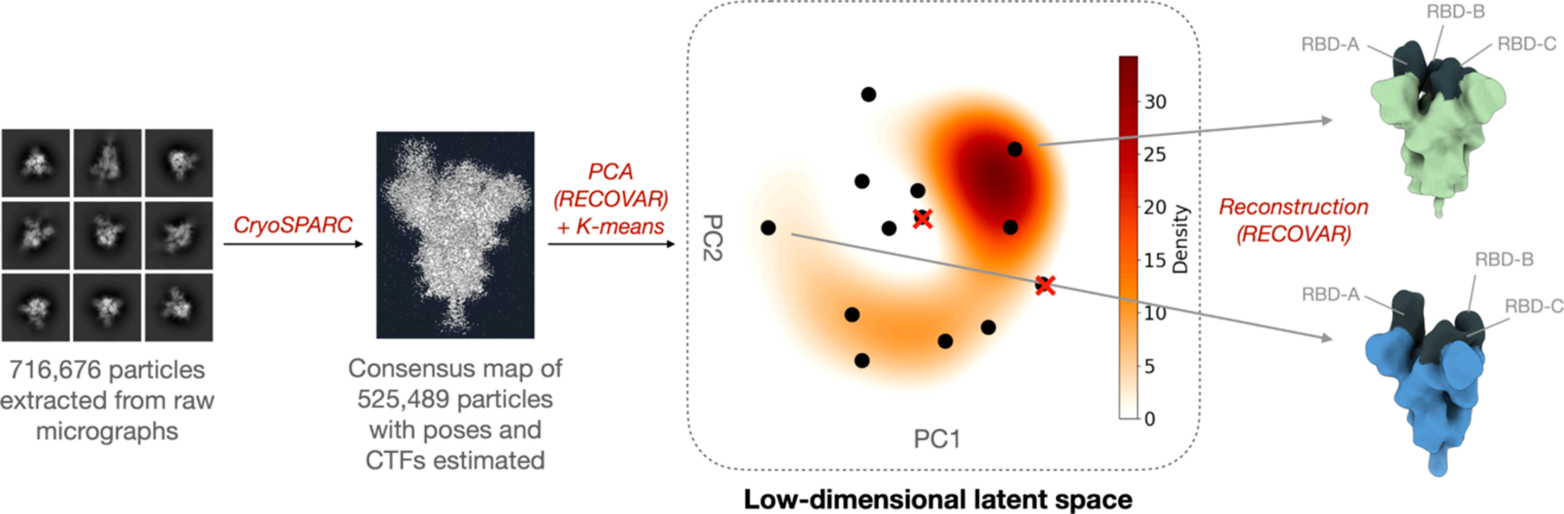
Computational pipeline for extracting domain-specific heterogeneity from cryo-EM 2D particles. 525489 2D particles with estimated contrast-transferring functions (CTFs) and poses from *CryoSPARC* (Punjani *et al.*, 2017 ▸) were projected to a low-dimensional latent space by principal component analysis (PCA) using *RECOVAR* (Gilles & Singer, 2025 ▸), from which 13 main volumes were found *via* k-means clustering. Volumes were constructed from them with *RECOVAR*, showing different up/down states of RBDs. Two volumes, corresponding to the centroids crossed out in the latent space, were excluded from the analysis since they were incomplete particles.

We performed a heterogeneity analysis on a data set of 716676 particles of the S protein–ACE2 complex using *RECOVAR* (Gilles & Singer, 2025 ▸) to distribute them in a latent space that linearly captures the heterogeneity of their associated 3D volume across the data. Upon applying the k-means clustering algorithm to this distribution, we identified 13 clusters of particles (see Fig. 2 ▸ and *Methods* section). In terms of representation, these clusters were relatively comparable in size, with ∼6–10% of the particles associated with each. As any point in latent space can be associated with a 3D volume, we then isolated the centroids of all the clusters to visualize the corresponding conformation of the spike protein. Upon inspection, we found two incomplete or damaged volumes that we further excluded from our analysis. Among the remaining 11 conformations, we noticed variations of the relative position of their RBDs, sug­gesting different configurations of up and down states. For ease of reference, we separately named each of the three RBDs as ‘RBD-A’, ‘RBD-B’, and ‘RBD-C’. RBD-A and RBD-B display different levels of opening, with greater intensity for RBD-A than RBD-B, and with RBD-C remaining in the down state in all 11 clusters.

### 2.2. Qu­anti­tative comparison of the RBD positions yields three principal RBD/ACE2 arrangements

**Figure 3:**
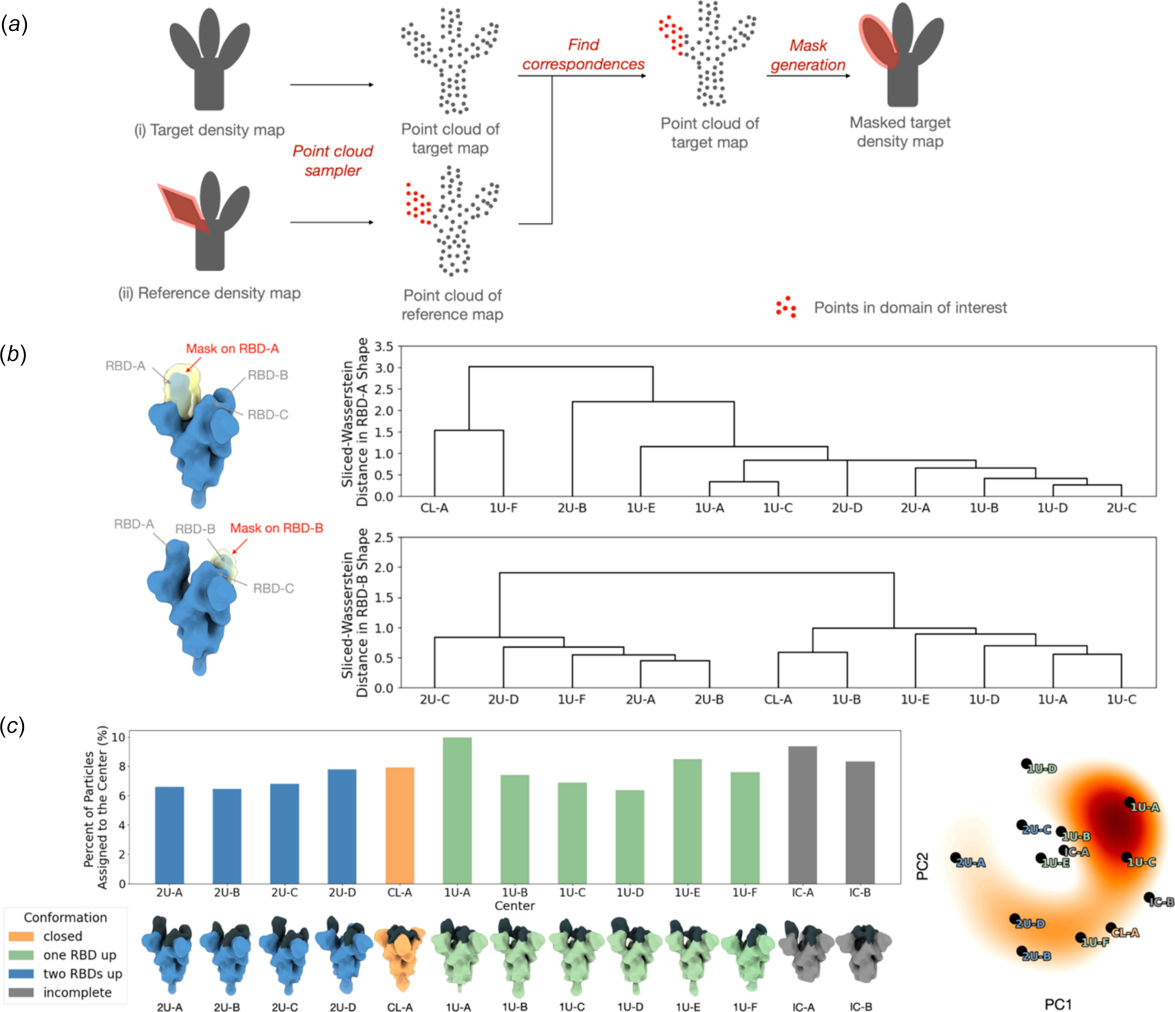
Clustering results on RBD-A and RBD-B shape reveal three distinct conformations of the main volumes according to their RBD/ACE2 arrangements. (*a*) The domain of inter­est of the density maps was masked through an automated protocol. Point clouds were generated using the topology representing network (TRN) sampler (Zhang *et al.*, 2021 ▸) from the input of (i) the target density map to be masked and (ii) a reference volume provided with the mask on the domain of inter­est. Points in the target volume corresponding to the points in the reference mask region were found *via* optimal transport. Gaussian functions centered at the transported points were summed up to form a mask covering the domain of inter­est in the target density map. (*b*) Masks were created on RBD-A and RBD-B of the 11 main volumes through the automated masking algorithm. Two clear clusters were formed in the hierarchical trees constructed with the pairwise sliced-Wasserstein distance (SWD) for both RBD-A and RBD-B masks. (*c*) The main volumes were classified into three RBD conformations based on hierarchical clustering results, namely, down, one-up, and two-up RBD states. Their positions in the plane formed by PC1 and PC2 in the embedding space, and the density maps constructed from their embeddings, are shown. Particles were evenly distributed among the main volumes.

In the next stage of the analysis, we sought to distinguish all-down, one-up, or two-up states among the 11 main conformational clusters. As the low resolution of the volumes did not allow inter­pretation of the maps using atomic models, we developed an automated masking procedure, based on optimal transport, to directly identify and measure the dif­ference in the positioning of the RBDs, which would also later be useful to analyze continuous paths between states [see Fig. 3 ▸(*a*) and *Methods* section]. The goal of this procedure is to guarantee consistency and mitigate bias in our inter­pretation of different states. After running the procedure to mask RBD-A, we obtained a set of pairwise sliced-Wasserstein distances (SWD) (Bonneel *et al.*, 2015 ▸) between the RBD-A masks of different volumes that allowed us to distinguish them using hierarchical clustering [Fig. 3 ▸(*b*)]. We found two main groups: the first contained two volumes showing RBD-A in the down state, while the other cluster had RBD-A in the up state. The same procedure on RBD-B yielded a first group of five volumes, with RBD-B in up states and the remaining six in down states [Fig. 3 ▸(*b*)]. We concluded that the 11 3D volumes extracted from the dataset showed three main RBD/ACE2 arrangements, with one all-closed, six one-up (plus one ACE2), and four two-up (plus two ACE2s) volumes [see Fig. 3 ▸(*c*) and Fig. S1 in the supporting information]. We called the only main volume with all RBDs in the down state ‘CL-A’, the six volumes with one RBD up ‘1U-A’, ‘1U-B’, ‘1U-C’, ‘1U-D’, ‘1U-E’, and ‘1U-F’, and the remaining four in two RBD up arrangements ‘2U-A’, ‘2U-B’, ‘2U-C’, and ‘2U-D’. Volume 1-F has a reversed RBD state, with RBD-B being in the up state and RBD-A in the down state. The two incomplete volumes that were not used in the analysis were named as ‘IC-A’ and ‘IC-B’.

### 2.3. Trajectories containing particles in inter­mediate states between one-RBD-up and two-RBD-up states show cooperativity between RBDs

**Figure 4:**
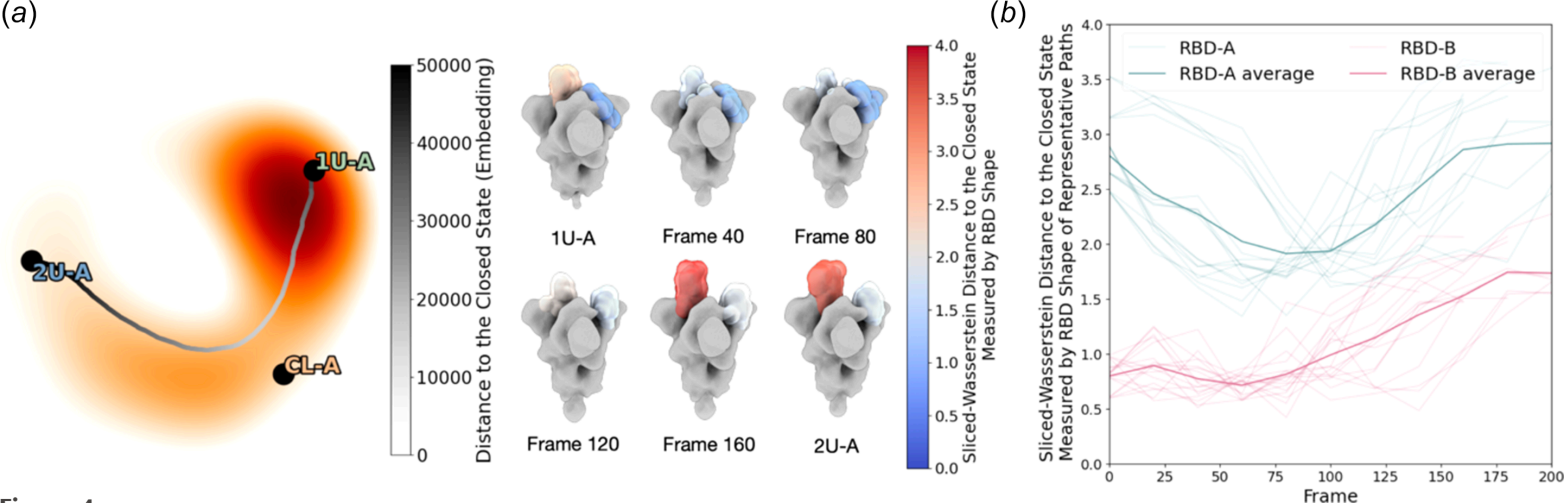
Representative trajectories between the one-RBD-up state and the two-RBD-up state indicate a significant preference to cooperative conformational changes. (*a*) The embeddings in the latent space and the density maps with the masks on RBD-A and RBD-B of the path connecting 1U-A and 2U-A indicate the existence of cooperativity between RBD-A and RBD-B. The masks were colored according to the sliced-Wasserstein distance (SWD) to the closed state. The conformation moved towards the closed state before transitioning to the two-RBD-up state according to the trajectory in the latent space and the SWD. (*b*) The line plot shows the SWD in RBD-A and RBD-B from CL-A of every 20 frames along all the representative trajectories. The weighted averages of the SWD in RBD-A and RBD-B of the representative paths were computed to provide an overview of the trend. A drop in RBD-A’s SWD, as well as its weighted average, was observed before the SWD of both RBD-A and RBD-B increased.

After extracting different main volumes and identifying the RBD arrangements, we took advantage of the representation of the particles in a latent space, to visualize the opening of RBD-B and the engagement of ACE2 between one-up and two-up states. To do so, we paired the main volumes with only RBD-A in the up state with volumes that had both RBD-A and RBD-B up, and computed for each pair (20 in total) the path joining states using the standard procedure in *RECOVAR*. We assigned for each path a score, reflecting how representative it is of the particles located in its proximity (see *Methods* section). Using this score, we then compared the 20 paths joining one-up to two-up states (Table 1 ▸), and found that three of them had a significantly lower score, sug­gesting that they were not representative of particles in the dataset. We then analyzed the 17 remaining representative paths showing the transition from one-up to two-up states. For each path, we ran the automated masking procedure again on the inter­mediate states of every 20 out of 200 frames, for a total of 11 states along the trajectory, to track the motion of RBD-A and RBD-B, and qu­anti­fied it using the Wasserstein distance with respect to CL-A, which had all RBDs closed. In the example shown in Fig. 4 ▸(*a*), we can observe that the trajectory significantly bent towards CL-A in the latent space, meaning that the S protein conformation gradually approached the all-down state in the middle of the transition. This is also confirmed by visualizing the RBD-A Wasserstein distance which dropped from the start to approximately frame 80 and then rose to the end. For RBD-B, we did not observe the same drop since it started in the down state, but the Wasserstein distance in­creased simultaneously as RBD-A, sug­gesting a joint conformational change in the two RBDs [see Fig. 4 ▸(*a*) and Video S1A in the supporting information]. We show in Fig. 4 ▸(*b*) that this pattern of ‘cooperativity’ is dominant in our dataset by computing a weighted mean of the representative paths (see *Methods* section for how to calculate weights). We also noted that in contrast, the three paths in the non-representative group showed a distinct pattern, where the distance of RBD-B from CL-A gradually increased, while the distance of RBD-A remained the same (see Fig. S2 and Video S1B in the supporting information for comparison).

To confirm that the transient states showing cooperative conformational changes between RBD-A and RBD-B did not just arise from the averages of the particles in the main states, we re-ran the pipeline with *RECOVAR* replaced by *cryoDRGN* (Zhong *et al.*, 2021 ▸), which is another heterogeneity analysis method that does not generate averaged volumes. The same cooperative mechanism was found by analysis with *cryoDRGN* (details are available in the supporting in­formation).

## 3. Discussion

In this article, we performed a heterogeneity analysis of a dataset of cryo-EM images of ACE2-bound spike proteins, with a spread in the up or down states of the RBD region. The choice of *RECOVAR* to perform our heterogeneity analysis, compared with other methods (Frank & Ourmazd, 2016 ▸; Nakane *et al.*, 2018 ▸; Chen & Ludtke, 2021 ▸; Punjani & Fleet, 2021 ▸; Zhong *et al.*, 2021 ▸; Vuillemot *et al.*, 2022 ▸; Punjani & Fleet, 2023 ▸) allowed us to preserve distances between volumes in the embedded space. In contrast to deep learning methods that are less robust in this regard (Edelberg & Lederman, 2023 ▸; Jeon *et al.*, 2024 ▸), *RECOVAR* is more suitable for modeling motions in RBDs considering the size of the domain, the spatial extent of its conformational change, and the consistency of the global shape of the RBD along its trajectory (*i.e.* as a compact folded domain). *RECOVAR* also presents the advantage of employing kernel regression to deconvolute uncertainty from the trajectory, so it is not blurred out through low SNR (see *Methods* section for details). For downstream analysis of the maps and paths generated by *RECOVAR*, we also needed to implement additional transport-based methods, for both masking and comparing subdomains across volumes and along trajectories. These new methods led to a more rigorous, qu­anti­fied, and high-throughput analysis of heterogeneity that extends beyond visual inspection of the averaged images.

With the proposed pipeline, we identified different conformations in SARS-CoV-2 S protein RBDs from cryo-EM images, including all down, one-up, and two-up RBD states, guided by the hierarchical analysis with masks. Only around 8% of particles were found to have all RBDs in the down state, consistent with our expectation that the presence of excess ACE2 would result in the transition of most of the RBDs to the up state (Lu *et al.*, 2020 ▸). No state with three RBDs up was discovered in this dataset, which is also expected considering the rareness of the conformation (Stalls *et al.*, 2022 ▸; Lee *et al.*, 2023 ▸). A recent heterogeneity analysis of the SARS-CoV-2 D614G spike (Herreros *et al.*, 2025 ▸), similar to the variant in this study, *i.e.* N501Y+D614G, reveals the same set of RBD arrangements. In essence, the discovery of these main states indicates that our pipeline was able to produce major SARS-CoV-2 RBD conformations consistent with previous studies.

The primary new finding from our work is the discovery of the paths describing the conformational changes between main states. Current descriptions of the opening of the spike protein with ACE2 engagement generally assume a pro­cess with sequential opening of RBDs (Benton *et al.*, 2020 ▸; Lu *et al.*, 2020 ▸). In contrast, our findings offer a different view, sug­gesting that the opening is a cooperative pro­cess. Although the resolution of the maps along the trajectories was not high enough for us to clearly delineate bound ACE2 in the density, our observations sug­gest the transition from the one-up to two-up states involves the disassociation of ACE2 in the midst of the transition.

The transient states obtained using *RECOVAR* may arise in principle from the averages of the particles in the main states without sufficient particles associated with the transient states connecting them. However, the representative paths captured from our dataset are not likely to be hallucinated volumes averaged from the main states, because the inter­mediate conformations are steered towards regions with high densities. We validated our findings by independently analyzing the same data set using *cryoDRGN*, which also generates a similar cooperative mechanism. In addition, a sanity check on the estimated poses was con­ducted to rigorously address the issue of (pseudo)symmetry, proving that the transient states of the cooperative behavior were unlikely to be the averages of the maps in the one-RBD-up state rotated by ±120° (see sup­porting information and Table S1). The heterogeneity analysis results obtained from our dataset thus support the existence of cooperativity between RBD-A and RBD-B, in contrast to a mechanism that implies sequential opening of each RBD protomer.

Many anti­bodies have been developed to neutralize the SARS-CoV-2 S protein, mostly by inter­acting with the epi­topes in open RBDs (Barnes *et al.*, 2020 ▸; Parray *et al.*, 2020 ▸; Wec *et al.*, 2020 ▸), but strategies that block transitions between conformations by binding to transient states remain underexplored. Targeting these structural motions is therefore an appealing prospect. Recent work in RBD dynamics (Tang *et al.*, 2025 ▸) sug­gests that RBDs of highly infective variants of SARS-CoV-2, such as Omicron, have a high affinity for the open state for ACE2, but may also favor states that lead to immune evasion.

There are multiple avenues to refine our current results. While the cFAR value (0.70) (Fig. S3 in the supporting in­formation) sug­gests that errors in pose estimation were not large enough to affect our main conclusion, jointly inferring or refining poses with respect to heterogeneity motion or by using a more static region of reference would allow us to decrease the error in our pose estimation step and potentially improve map resolution. To do so, one approach to consider for future studies is to iteratively refine poses and heterogeneity. We also did not consider here whether ACE2 proteins were truly attached to RBDs, which will require analysis of a much larger dataset and methods to segment ACE2 from RBD regions. Another challenge is that, given the nature of the cryo-EM experiment, the trajectories generated by heterogeneity analysis are derived from an analysis of the conformational ensemble as a single point in time when the sample is plunge-frozen. Finally, it will be important to evaluate the generalizability of our results to other SARS-CoV-2 mutants where it is already known that RBD dynamics is impacted (Barton *et al.*, 2021 ▸; da Costa *et al.*, 2022 ▸; Valério *et al.*, 2022 ▸; Xue *et al.*, 2024 ▸), and to other protein complexes where there is evidence of large-scale quaternary changes in conformation.

Using a reliable benchmark is an important aspect of con­ducting heterogeneity analysis in projection images recorded using cryo-EM, in part because it is difficult to establish ‘ground truth’ structures with confidence, and because there is no obvious metric to evaluate the reliability of the heterogeneity analysis. While synthetic cryo-EM datasets can be used as a possible benchmark (Jeon *et al.*, 2024 ▸), the performance of the methods can be significantly affected by the type of motion that is being analyzed. It is therefore important to execute multiple types of heterogeneity analysis methods in order not to be biased by a single model. In this study, we focused on the results from *RECOVAR*, which fits our tasks well as a linear subspace approach, but we also verified its reliability by comparing the results with *cryoDRGN. cryoDRGN*, with a variational autoencoder (VAE) structure, is based on a completely different framework from *RECOVAR*, and thus serves as a good independent benchmark model. A recent study has started to systematically evaluate the similarities and dif­ferences between those heterogeneity methods by placing embeddings from different methods into a consensus space (Herreros *et al.*, 2025 ▸). In the future, it will be inter­esting to expand the application of different heterogeneity methods to a larger range of datasets.

## 4. Methods

### 4.1. Image pro­cessing and pose estimation

The cryo-EM dataset used in our analysis was previously used to obtain structures of the N501Y/D614G mutant deposited as PDB ID 7sy1 (EMDB-25513) and PDB ID 7sy2 (EMDB-25514) (Mannar *et al.*, 2021 ▸). Cryo-EM samples were prepared at pH 8.0 and imaged using a 300 kV Titan Krios G4 transmission electron microscope (Thermo Fisher Scientific) equipped with a Falcon4 direct electron detector in electron event registration (EER) mode. Movies were collected at 155000× magnification (physical pixel size 0.5 Å). Raw cryo-EM micrographs were pro­cessed and poses of extracted particles in the consensus model were estimated with *Cryo­SPARC* (version 4.6.0; Punjani *et al.*, 2017 ▸), as shown in Fig. S3. Prepro­cessing started with motion correction, fol­lowed by patch contrast transfer function (CTF) estimation. Micrographs with the CTF fit resolution between 2.347 and 10 Å, the relative ice thickness between 0.998 and 2, and the defocus tilt angle between 0 and 20° were selected in the curate exposure. After the Blob picking with the diameter ranging from 120 to 220 Å and a minimum separation of 0.5, the inspect picks with power between 70 and 150, particle extraction with a box size of 800 px, and a brief cleaning by 2D classification with 40 EM iterations, five final iterations, and a batch size of 200, from which clear junk particles were removed in the data, we were left with 716676 particles. Particles were down-sampled to 400 px before the pose estimation.

In the first step of the pose estimation, particles were classified in 2D. We used 217728 particles of different views with the highest quality to construct three *ab initio* models. Then 22 classes containing 624143 particles that were not junk particles were used to refine three heterogeneous models starting with the *ab initio* models from the last step. Unlike the regular cryo-EM map construction, all the particles resembling the SARS-CoV-2 S protein were included, even those with low quality, because we were also inter­ested in transient states which were less representative in the dataset and thus had low quality when generating 2D classes. Any junk particles included in this step are expected to be separated out in the heterogeneity analysis. After the heterogeneity refinement, 525489 particles assigned to the map of an intact SARS-CoV-2 S protein were retained, with final poses estimated using a homogeneous refinement step. The particles were further down-sampled to 128 px before embedding.

### 4.2. Heterogeneity analysis with *RECOVAR*

We performed heterogeneity analysis on the 525489 pro­cessed 2D particles using *RECOVAR* (Gilles & Singer, 2025 ▸), with the number of dimensions for the latent space equal to four. After obtaining the embeddings for the 2D particles, we clustered the particles using k-means on all the embedded points except 10% of the points with the highest embedding uncertainty, calculated as the 2-norm of the 4 × 4 covariance matrix of each embedding. The number of clusters was determined by the ‘elbow rule’ and was set to 13. Paths were generated between the main volumes of inter­est using the path discovery method in *RECOVAR*, which computed the trajectory with the highest deconvolved density (which was again computed after excluding 10% of the points with the highest embedding uncertainty) between the given start and end points by solving an eikonal equation. Paths were represented by a series of points. 3D density maps corresponding to the cluster centroids and the points along the trajectories were generated by kernel regression in *RECOVAR*.

### 4.3. Point cloud sampling and automated masking

We developed a procedure to automate masking of domains of inter­est (*e.g.* RBDs) in density maps. Before masking, we cleaned the maps by down-sampling with a bin size of 2 and removing noise ‘dust’ with the tool ‘Hide Dust’ with size limit set to 100 voxels from the raw maps using *UCSF ChimeraX* (Meng *et al.*, 2023 ▸). The algorithm for automated mask generation requires a reference map with the domain of inter­est masked as input. The topology representing network (TRN) method [see Zhang *et al.* (2021 ▸) or Tajmir Riahi *et al.* (2025 ▸) for similar implementation] was used to create a point cloud from the reference map, with the probability distribution following normalized voxel intensities. To achieve this, 1000 points were first sampled with replacement from the voxel coordinates, using probabilities weighted by voxel intensities. An iterative diffusion pro­cess was then applied. In each iteration, a point was sampled from the voxel coordinates, again based on the probability proportional to voxel intensities, and the 1000 points were moved towards the point. The reason we need diffusion besides the initial sampling is that 1000 points are not sufficient to converge to the desired probability distribution, while sampling an excessive number of points could lead to a substantial increase in computational time for solving the optimal transport problem. Points ending up in the masked region were recorded. We could easily find point correspondences between the reference map and the target map *via* solving an entropic regularization optimal transport problem (Cuturi, 2013 ▸), where each point of the source map found a corresponding point on the target by maximizing the transport map. We tried a variety of values of the regularization parameter between 1e-3 and 10 on small samples, and decided to use ω = 5 for the main volume and path mask generation, since at that value we got least noise, as well as reasonable coverage of RBDs.

The mask was then generated by summing up Gaussian functions centered at these points as follows: 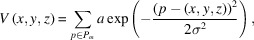
where *V*(*x*, *y*, *z*) is the voxel intensity at coordinate (*x*, *y*, *z*) of the mask we would like to create. *P_m_* is the set of point coordinates in the target map corresponding to the points in the masked region of the reference map. We took *a* = σ = 1. There is a recent study using k-means instead of TRN to fit a point cloud (Raghu *et al.*, 2025 ▸). A comparison was made by replacing TRN with weighted k-means clustering to compute 1000 centroids of the voxel coordinates with weights to be proportional to the voxel intensities. All the other steps or parameters were kept the same. However, no significant dif­ference was noticed between the masks created by TRN and k-means, as indicated by Fig. S4 (see supporting information), and TRN requires significantly less time to compute point clouds. We therefore adopted TRN for our mask generation algorithm.

In our dataset, we used manual masks generated on 1U-A’s RBD-A and 2U-A’s RBD-B with *UCSFChimeraX* (Meng *et al.*, 2023 ▸) as reference masks for RBD-A and RBD-B, res­pectively, since their signals were strong and make it easy to create masks manually. All the masks showed a reasonable shape and placement upon visual inspection (see Fig. S5 in the supporting information for masks of the main volumes).

### 4.4. Wasserstein distance

After obtaining masks around regions of inter­est, conformational changes were then directly qu­anti­fied *via* the 2-sliced-Wasserstein distance (Bonneel *et al.*, 2015 ▸) between the masks, where samples are voxel coordinates, *i.e.* {(*i*, *j*, *k*) ∣ *i*, *j*, *k* ∈ [*N*]}, with *N* being the grid dimension and weights are voxel intensities. The 2-sliced-Wasserstein distance was com­puted by Monte Carlo approximation. We did not compute the full Wasserstein distance, whose cost matrix is required to hold *N*^6^ entries, leading to extremely high computational costs.

To quan­ti­ta­tively infer the states of the main volumes in our dataset, the pairwise 2-sliced-Wasserstein distance was com­puted between the RBD-A/RBD-B masks generated for the main volumes, from which hierarchical clustering was done by the unweighted pair group method with arithmetic mean (UPGMA). To assess the conformational changes along the paths, the Wasserstein distance was computed between the RBD-A/RBD-B masks of every 20 frames and the RBD-A/RBD-B masks of the CL-A density map with all three RBDs closed, as an estimation of the degree of opening.

### 4.5. Path analysis

To analyze a collection of paths (*T_i_*) inferred by *RECOVAR*, each path being represented by a series of points *p_ij_*, we implemented the following procedure to evaluate their agreement with the data. First, we defined a ‘representativity score’ for each path *T_i_*, computed as: 
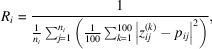
where *n_i_* is the number of points along path *T_i_*, *p_ij_*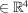 is the embedded point corresponding to frame *j* of path *T_i_*, and 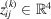 is the *k*th point in the ranked embedded points (after excluding 10% of the points with the highest embedding uncertainty) by distance to *p_ij_*, *i.e.*
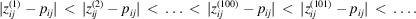
In other words, for each point we found 100 points that were closest to it in the latent space and computed its average squared distance to these 100 points, which tends to be low if the density around the embedded point is high. This was then averaged among all the points along the paths. We took its inverse to obtain the final representativity score. We also computed representativity scores after removing the first and last quarter of the paths to focus on the middle part and found it did not affect our main results and conclusion (see Table 1 ▸). In addition, to generate a weighted mean over all the representative trajectories [see Figs. 4 ▸(*b*) and S2(*b*)], we assigned to each embedded point *p_ij_* the weight value 
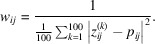


## Supplementary Material

Video S1A. DOI: 10.1107/S2052252526004513/hi5657sup1.mov

Video S1B. DOI: 10.1107/S2052252526004513/hi5657sup2.mov

Video S2. DOI: 10.1107/S2052252526004513/hi5657sup3.mov

Supporting information. DOI: 10.1107/S2052252526004513/hi5657sup4.pdf

## Figures and Tables

**Table 1:** Representativity scores (1e-7) of the paths transitioning from the main volumes with one RBD up to the volumes in the two-RBD-up state indicate different levels of agreement with the data The values in the parentheses were calculated after excluding the first and last quarter of the paths to eliminate effects from the start and end points.

		Two RBDs up			
From/To		2U-A	2U-B	2U-C	2U-D
One RBD up	1U-A	0.93110 (1.01618)	0.95613 (1.05137)	0.96594 (0.98021)	1.02154 (1.05713)
	1U-B	0.87763 (1.01469)	0.88062 (0.99523)	0.90477 (0.99259)	0.92812 (0.98921)
	1U-C	0.91288 (1.04330)	0.93580 (1.08111)	0.94607 (1.03215)	0.98387 (1.09456)
	1U-D	0.73040 (0.77848)*	0.94874 (1.11138)	0.94875 (1.05369)	0.98983 (1.11903)
	1U-E	0.70635 (0.73911)*	0.70831 (0.71726)*	0.94203 (1.03577)	0.98461 (1.09912)

Note: (*) low representativity.

## References

[bb1] Barnes, C. O., West, A. P., Huey-Tubman, K. E., Hoffmann, M. A. G., Sharaf, N. G., Hoffman, P. R., Koranda, N., Gristick, H. B., Gaebler, C., Muecksch, F., Lorenzi, J. C. C., Finkin, S., Hägglöf, T., Hurley, A., Millard, K. G., Weisblum, Y., Schmidt, F., Hatziioannou, T., Bieniasz, P. D., Caskey, M., Robbiani, D. F., Nussenzweig, M. C. & Bjorkman, P. J. (2020). *Cell* **182**, 828–842. 10.1016/j.cell.2020.06.025 PMC7311918 32645326

[bb2] Barton, M. I., MacGowan, S. A., Kutuzov, M. A., Dushek, O., Barton, G. J. & van der Merwe, P. A. (2021). *eLife* **10**, e70658. 10.7554/eLife.70658 PMC8480977 34435953

[bb3] Benton, D. J., Wrobel, A. G., Xu, P., Roustan, C., Martin, S. R., Rosenthal, P. B., Skehel, J. J. & Gamblin, S. J. (2020). *Nature* **588**, 327–330. 10.1038/s41586-020-2772-0 PMC7116727 32942285

[bb4] Bonneel, N., Rabin, J., Peyré, G. & Pfister, H. (2015). *J. Math. Imaging Vis.* **51**, 22–45.

[bb5] Cascella, M., Rajnik, M., Aleem, A., Dulebohn, S. C. & Di Napoli, R. (2025). *StatPearls*. Treasure Island (FL): StatPearls Publishing. 32150360

[bb6] Cerutti, G., Guo, Y., Liu, L., Liu, L., Zhang, Z., Luo, Y., Huang, Y., Wang, H. H., Ho, D. D., Sheng, Z. & Shapiro, L. (2022). *Cell. Rep.* **38**, 110428. 10.1016/j.celrep.2022.110428 PMC8818377 35172173

[bb7] Chen, M. & Ludtke, S. J. (2021). *Nat. Methods* **18**, 930–936. 10.1038/s41592-021-01220-5 PMC8363932 34326541

[bb8] Chen, Y., Zhao, X., Zhou, H., Zhu, H., Jiang, S. & Wang, P. (2023). *Nat. Rev. Immunol.* **23**, 189–199. 10.1038/s41577-022-00784-3 PMC9514166 36168054

[bb9] Cuturi, M. (2013). *Adv. Neural Inf. Process. Syst.* **26**, 2292–2300.

[bb10] da Costa, C. H. S., de Freitas, C. A. B., Alves, C. N. & Lameira, J. (2022). *Sci. Rep.* **12**, 8540. 10.1038/s41598-022-12479-9 PMC9121086 35595778

[bb11] Duan, L., Zheng, Q., Zhang, H., Niu, Y., Lou, Y. & Wang, H. (2020). *Front. Immunol.* **11**, 576622. 10.3389/fimmu.2020.576622 PMC7575906 33117378

[bb12] Edelberg, D. G. & Lederman, R. R. (2023). *arXiv*, 2303.07487.

[bb13] Frank, J. & Ourmazd, A. (2016). *Methods* **100**, 61–67. 10.1016/j.ymeth.2016.02.007 PMC4848141 26884261

[bb14] Gilles, M. A. & Singer, A. (2025). *Proc. Natl Acad. Sci. USA* **122**, e2419140122. 10.1073/pnas.2419140122 PMC11892586 40009640

[bb15] Henderson, R., Edwards, R. J., Mansouri, K., Janowska, K., Stalls, V., Gobeil, S. M. C., Kopp, M., Li, D., Parks, R., Hsu, A. L., Borgnia, M. J., Haynes, B. F. & Acharya, P. (2020). *Nat. Struct. Mol. Biol.* **27**, 925–933. 10.1038/s41594-020-0479-4 PMC8581954 32699321

[bb16] Herreros, D., Perez Mata, C., Sanchez Sorzano, C. O. & Carazo, J. M. (2025). *Nat. Methods* **22**, 2118–2126. 10.1038/s41592-025-02841-w PMC12510870 40999098

[bb17] Huang, Y., Yang, C., Xu, X., Xu, W. & Liu, S. (2020). *Acta Pharmacol. Sin.* **41**, 1141–1149. 10.1038/s41401-020-0485-4 PMC7396720 32747721

[bb18] Jeon, M., Raghu, R., Astore, M., Woollard, G., Feathers, R., Kaz, A., Hanson, S. M., Cossio, P. & Zhong, E. D. (2024). *arXiv*, 2408.05526.

[bb19] Ke, Z., Oton, J., Qu, K., Cortese, M., Zila, V., McKeane, L., Nakane, T., Zivanov, J., Neufeldt, C. J., Cerikan, B., Lu, J. M., Peukes, J., Xiong, X., Kräusslich, H.-G., Scheres, S. H. W., Bartenschlager, R. & Briggs, J. A. G. (2020). *Nature* **588**, 498–502. 10.1038/s41586-020-2665-2 PMC7116492 32805734

[bb20] Lan, J., Ge, J., Yu, J., Shan, S., Zhou, H., Fan, S., Zhang, Q., Shi, X., Wang, Q., Zhang, L. & Wang, X. (2020). *Nature* **581**, 215–220. 10.1038/s41586-020-2180-5 32225176

[bb21] Lee, M., Major, M. & Hong, H. (2023). *Int. J. Mol. Sci.* **24**, 3774. 10.3390/ijms24043774 PMC9967551 36835186

[bb22] Lu, M., Uchil, P. D., Li, W., Zheng, D., Terry, D. S., Gorman, J., Shi, W., Zhang, B., Zhou, T., Ding, S., Gasser, R., Prévost, J., Beaudoin-Bussières, G., Anand, S. P., Laumaea, A., Grover, J. R., Liu, L., Ho, D. D., Mascola, J. R., Finzi, A., Kwong, P. D., Blanchard, S. C. & Mothes, W. (2020). *Cell Host Microbe* **28**, 880–891. 10.1016/j.chom.2020.11.001 PMC7664471 33242391

[bb23] Mannar, D., Saville, J. W., Poloni, C., Zhu, X., Bezeruk, A., Tidey, K., Ahmed, S., Tuttle, K. S., Vahdatihassani, F., Cholak, S., Cook, L., Steiner, T. S. & Subramaniam, S. (2024). *Nat. Commun.* **15**, 1854. 10.1038/s41467-024-46104-2 PMC10904792 38424106

[bb24] Mannar, D., Saville, J. W., Zhu, X., Srivastava, S. S., Berezuk, A. M., Tuttle, K. S., Marquez, A. C., Sekirov, I. & Subramaniam, S. (2022). *Science* **375**, 760–764. 10.1126/science.abn7760 PMC9799367 35050643

[bb25] Mannar, D., Saville, J. W., Zhu, X., Srivastava, S. S., Berezuk, A. M., Zhou, S., Tuttle, K. S., Kim, A., Li, W., Dimitrov, D. S. & Subramaniam, S. (2021). *Cell. Rep.* **37**, 110156. 10.1016/j.celrep.2021.110156 PMC8642162 34914928

[bb26] Meng, E. C., Goddard, T. D., Pettersen, E. F., Couch, G. S., Pearson, Z. J., Morris, J. H. & Ferrin, T. E. (2023). *Protein Sci.* **32**, e4792. 10.1002/pro.4792 PMC10588335 37774136

[bb27] Min, L. & Sun, Q. (2021). *Front. Mol. Biosci.* **8**, 671633. 10.3389/fmolb.2021.671633 PMC8100443 33968996

[bb28] Nakane, T., Kimanius, D., Lindahl, E. & Scheres, S. H. (2018). *eLife* **7**, e36861. 10.7554/eLife.36861 PMC6005684 29856314

[bb29] Parray, H. A., Chiranjivi, A. K., Asthana, S., Yadav, N., Shrivastava, T., Mani, S., Sharma, C., Vishwakarma, P., Das, S., Pindari, K., Sinha, S., Samal, S., Ahmed, S. & Kumar, R. (2020). *J. Biol. Chem.* **295**, 12814–12821. 10.1074/jbc.AC120.014918 PMC7476711 32727845

[bb30] Punjani, A. & Fleet, D. J. (2021). *J. Struct. Biol.* **213**, 107702. 10.1016/j.jsb.2021.107702 33582281

[bb31] Punjani, A. & Fleet, D. J. (2023). *Nat. Methods* **20**, 860–870. 10.1038/s41592-023-01853-8 PMC10250194 37169929

[bb32] Punjani, A., Rubinstein, J. L., Fleet, D. J. & Brubaker, M. A. (2017). *Nat. Methods* **14**, 290–296. 10.1038/nmeth.4169 28165473

[bb33] Raghu, R., Levy, A., Wetzstein, G. & Zhong, E. D. (2025). *arXiv*, 2506.04490.

[bb34] Salamanna, F., Maglio, M., Landini, M. P. & Fini, M. (2020). *Front. Med.* **7**, 594495. 10.3389/fmed.2020.594495 PMC7744810 33344479

[bb35] Saville, J. W., Mannar, D., Zhu, X., Srivastava, S. S., Berezuk, A. M., Demers, J.-P., Zhou, S., Tuttle, K. S., Sekirov, I., Kim, A., Li, W., Dimitrov, D. S. & Subramaniam, S. (2022). *Nat. Commun.* **13**, 742. 10.1038/s41467-022-28324-6 PMC8826856 35136050

[bb36] Stalls, V., Lindenberger, J., Gobeil, S. M.-C., Henderson, R., Parks, R., Barr, M., Deyton, M., Martin, M., Janowska, K., Huang, X., May, A., Speakman, M., Beaudoin, E., Kraft, B., Lu, X., Edwards, R. J., Eaton, A., Montefiori, D. C., Williams, W. B., Saunders, K. O., Wiehe, K., Haynes, B. F. & Acharya, P. (2022). *Cell. Rep.* **39**, 111009. 10.1016/j.celrep.2022.111009 PMC9174147 35732171

[bb37] Tajmir Riahi, A., Zhang, C., Condon, A., Chen, J. & Dao Duc, K. (2025). *PRX Life* **3**, 023003.

[bb38] Tang, C., Lupala, C. S., Wang, D., Li, X., Tang, L.-H. & Li, X. (2025). *Int. J. Mol. Sci.* **26**, 3776. 10.3390/ijms26083776 PMC12027596 40332432

[bb39] Valério, M., Borges-Araújo, L., Melo, M. N., Lousa, D. & Soares, C. M. (2022). *Front. Med. Technol.* **4**, 1009451. 10.3389/fmedt.2022.1009451 PMC9581196 36277437

[bb40] Vuillemot, R., Miyashita, O., Tama, F., Rouiller, I. & Jonic, S. (2022). *J. Mol. Biol.* **434**, 167483. 10.1016/j.jmb.2022.167483 35150654

[bb41] Walls, A. C., Park, Y.-J., Tortorici, M. A., Wall, A., McGuire, A. T. & Veesler, D. (2020). *Cell* **181**, 281–292.e6. 10.1016/j.cell.2020.02.058 PMC7102599 32155444

[bb42] Wec, A. Z., Wrapp, D., Herbert, A. S., Maurer, D. P., Haslwanter, D., Sakharkar, M., Jangra, R. K., Dieterle, M. E., Lilov, A., Huang, D., Tse, L. V., Johnson, N. V., Hsieh, C.-L., Wang, N., Nett, J. H., Champney, E., Burnina, I., Brown, M., Lin, S., Sinclair, M., Johnson, C., Pudi, S., Bortz, R., Wirchnianski, A. S., Laudermilch, E., Florez, C., Fels, J. M., O’Brien, C. M., Graham, B. S., Nemazee, D., Burton, D. R., Baric, R. S., Voss, J. E., Chandran, K., Dye, J. M., McLellan, J. S. & Walker, L. M. (2020). *Science* **369**, 731–736. 10.1126/science.abc7424 PMC7299279 32540900

[bb43] World Health Organization (2025). *Number of COVID-19 deaths reported to WHO*. https://data.who.int/dashboards/covid19/deaths.

[bb44] Wu, F., Zhao, S., Yu, B., Chen, Y.-M., Wang, W., Song, Z.-G., Hu, Y., Tao, Z.-W., Tian, J.-H., Pei, Y.-Y., Yuan, M.-L., Zhang, Y.-L., Dai, F.-H., Liu, Y., Wang, Q.-M., Zheng, J.-J., Xu, L., Holmes, E. C. & Zhang, Y.-Z. (2020). *Nature* **579**, 265–269. 10.1038/s41586-020-2008-3 PMC7094943 32015508

[bb45] Xue, S., Han, Y., Wu, F. & Wang, Q. (2024). *Protein Cell* **15**, 403–418. 10.1093/procel/pwae007 PMC11131022 38442025

[bb46] Yuan, M., Liu, H., Wu, N. C. & Wilson, I. A. (2021). *Biochem. Biophys. Res. Commun.* **538**, 192–203. 10.1016/j.bbrc.2020.10.012 PMC7547570 33069360

[bb47] Zhang, Y., Krieger, J., Mikulska-Ruminska, K., Kaynak, B., Sorzano, C. O. S., Carazo, J.-M., Xing, J. & Bahar, I. (2021). *Prog. Biophys. Mol. Biol.* **160**, 104–120. 10.1016/j.pbiomolbio.2020.08.006 PMC7914283 32866476

[bb48] Zhao, Z., Zhou, J., Tian, M., Huang, M., Liu, S., Xie, Y., Han, P., Bai, C., Han, P., Zheng, A., Fu, L., Gao, Y., Peng, Q., Li, Y., Chai, Y., Zhang, Z., Zhao, X., Song, H., Qi, J., Wang, Q., Wang, P. & Gao, G. F. (2022). *Nat. Commun.* **13**, 4958. 10.1038/s41467-022-32665-7 PMC9399999 36002453

[bb49] Zhong, E. D., Bepler, T., Berger, B. & Davis, J. H. (2021). *Nat. Methods* **18**, 176–185. 10.1038/s41592-020-01049-4 PMC8183613 33542510

[bb50] Zhu, X., Mannar, D., Srivastava, S. S., Berezuk, A. M., Demers, J.-P., Saville, J. W., Leopold, K., Li, W., Dimitrov, D. S., Tuttle, K. S., Zhou, S., Chittori, S. & Subramaniam, S. (2021). *PLOS Biol.* **19**, e3001237. 10.1371/journal.pbio.3001237 PMC8112707 33914735

