# Supplementary material for: Cryo-EM analysis of cooperative conformational changes in the SARS-CoV-2 spike protein trimer
Source: IUCrJ. 2026 Jun 25;13(Pt 4):385–94. doi: 10.1107/S2052252526004513 (PMC13324612; doi:10.1107/S2052252526004513)
Supplement: Supplementary file 4 [file m-13-00385-sup4.pdf]

IUCrJ

Volume 13 (2026)

Supporting information for article:

Cryo-EM analysis of cooperative conformational changes in the SARS-CoV-2 spike protein trimer

Qiyu Wang, Spencer Cholak, Geoffrey Woollard, Sriram Subramaniam and Khanh Dao Duc

S1. Orientation diagnostics from pose estimation

To check if significant errors existed in the pose estimation, we ran an Orientation Diagnostics job in CryoSPARC to get the conical Fourier Shell Correlation Area Ratio (cFAR). We then examined whether pose estimation was affected by C3 (pseudo-)symmetry of SARS-CoV-2 spike particles, which can result in estimated poses rotated by ±120° around the z-axis from true poses. We ran another five homogeneous refinement jobs on the 525,489 particles with the same parameters. We extracted azimuths (i.e. rotation angles around the z-axis) of the particles in each replicated run and computed their difference with the azimuths of the poses from the main run. We examined the proportion of particles with azimuth angles rotated by ±120°, which is expected to be large if the problem caused by (pseudo-)symmetry truly exists. We also investigated the proportion of particles without significant changes in azimuths. We allowed for an uncertainty of 10°, meaning that the percentage falling in [-130°, -110°] ∪ [110°, 130°] was computed for the former, and the percentage falling in [-10°, 10°] was calculated for the latter (see Supplementary Table S1). We observed only ~3% of particles switched symmetry groups in each run, which is unlikely to bias our results.

S2. Benchmark with cryoDRGN

To independently validate our findings, we re-ran the pipeline to generate trajectories after replacing RECOVAR by cryoDRGN. Unlike RECOVAR, cryoDRGN has a nonlinear mapping of heterogeneity to volume, without source distribution estimation via deconvolution. On the other hand, all the points along the paths output by cryoDRGN come from the embeddings of the input particles, from which volumes are constructed by a decoder, thus not having the problem of artificially generated density maps from the averaged main states. CryoDRGN therefore serves as an effective means to assess the robustness of the heterogeneity analysis results from RECOVAR.

The same set of 525,489 2D particles along with their estimated poses and CTFs were input into cryoDRGN. A 4D latent space and a train epoch number of 20 were applied. Without the estimated covariance and mean of the conformational states as in RECOVAR, which were required for path discovery by deconvolved densities, cryoDRGN aims to find the shortest path along the nearest-neighbor graph created from the latent space embeddings connecting a series of anchor points. Therefore, unlike RECOVAR where we could specify the start and

end points and generate multiple paths connecting different main volumes, cryoDRGN outputs one trajectory only describing the overall conformational change. We generated the cryoDRGN trajectory by setting the thirteen centroids computed from k-means clustering (Supplementary Figure S6) as the anchor points. Density maps were generated by putting embeddings corresponding to transient states along the trajectory into the pre-trained decoder. We adopted the same RBD naming strategy as used for the main volumes found with the pipeline with RECOVAR and called the three RBDs 'RBD-A', 'RBD-B 'and 'RBD-C', where RBD-C remained closed, and RBD-A and RBD-B showed various degree of opening. The same masking procedure as shown in Figure 3A was applied with manual masks on center 3's RBD-A and center 9's RBD-B as reference.

Following the same strategy as before, the SWD in masked RBD-A/RBD-B along the trajectory with center 2's RBD-A and center 0's RBD-B (which were the most clear and closest to the down position) was computed as the indicator of RBD-A/RBD-B's opening degree. Results are present in Figure S7A. No significant changes in RBD-B were found until frame 250, starting from which RBD-B's SWD with the closed state increased dramatically. It is worth noting that there was also a drop in the SWD of RBD-A to the closed state from frame 160 to 240, right before the SWD in RBD-B started to rise. The SWD of RBD-A then increased together with RBD-B's. This pattern is consistent with representative trajectories computed via the pipeline with RECOVAR. Figure S7B highlights cooperative conformational changes between frame 160 and frame 260. Video S2 offers a visualization of the entire cryoDRGN trajectory.

S3. Comparison of cryoDRGN with RECOVAR

Overall, the trajectory results from the pipeline using cryoDRGN agree with RECOVAR, in the sense that the only conformational change in RBD-B along the cryoDRGN trajectory was accompanied by changes in RBD-A as demonstrated by the results from RECOVAR, supporting the conclusion from our analysis.

Despite the consistency of the results between cryoDRGN and RECOVAR, there are specific advantages to our approach based on using RECOVAR. First, it is more convenient to use RECOVAR to study transitions between specific configurations in our case since it allows us to set start and end points. Moreover, when inspecting movies, we noticed that the cryoDRGN trajectory was less smooth compared with RECOVAR paths, perhaps due to the

non-linear decoder, or the lack of deconvolution to account for per-image latent variable uncertainty, observed in previous studies (Jeon *et al.*, 2024).

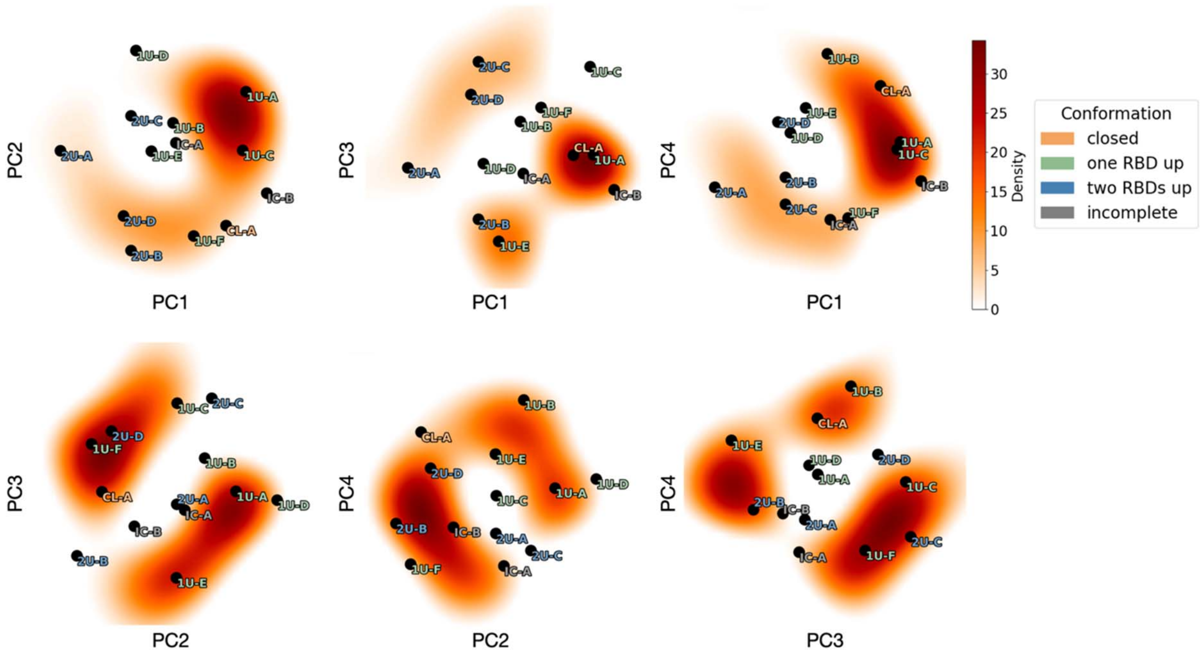

Figure S1 Classified centroids visualized in the latent space formed by pairs of principal components (PCs).

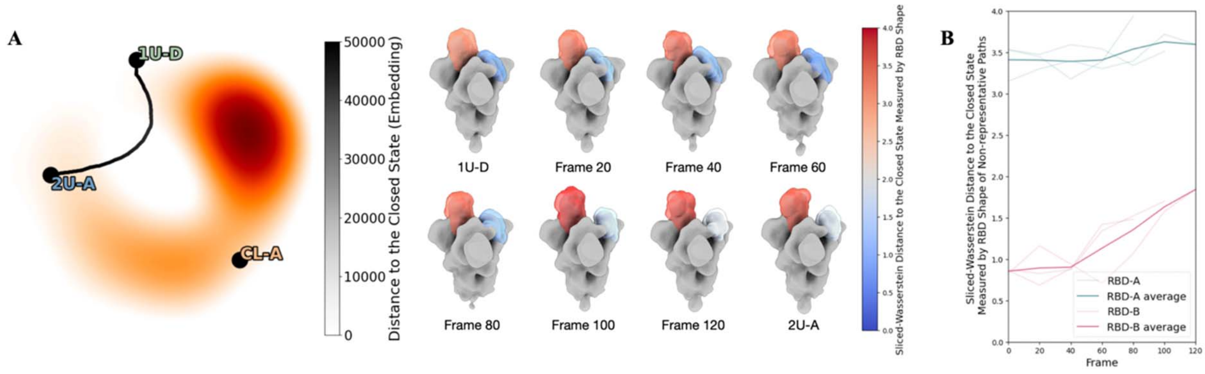

Figure S2 The transition from 1U-D with one RBD up to 2U-A with two RBDs up follows sequential opening instead of cooperative opening. (A) There is no obvious change in RBD-A's sliced-Wasserstein distance (SWD) to the closed state during the entire opening process of RBD-B. (B) The flat profile of the SWD in RBD-A of the three non-representative paths implies sequential opening.

Table S1 Percentages of particles in replicated runs with ~ ±120° rotations or no significant changes in the azimuths from reference poses indicate the robustness of the pose estimation to (pseudo-)symmetry. Five replicated homogeneous refinement jobs were performed. The percentage of particles rotated by ~ ±120° for each run was calculated as the proportion of particles whose difference in the azimuths with the poses input into RECOVAR falling in [-130°, -110°] ∪ [110°, 130°], while the percentage without significant difference is the proportion falling into [-10°, 10°]. Percentages for good particles were computed after excluding 10% of particles with the highest embedding uncertainty. Averaged percentages of the five runs were calculated. The small fraction of rotated particles and the large proportion showing no noticeable change in azimuths indicate that the pose estimation is robust despite the potential (pseudo-)symmetry problem of the SARS-CoV-2 S protein.

	**All particles**		**Good particles**	
	**Rotated by ~ ±120°**	**No significant difference**	**Rotated by ~ ±120°**	**No significant difference**
**Run 1**	3.66	91.38	3.67	91.39
**Run 2**	3.40	92.19	3.39	92.19
**Run 3**	3.60	91.43	3.60	91.44
**Run 4**	3.73	91.60	3.73	91.62
**Run 5**	3.79	91.04	3.80	91.04
**Average**	3.64	91.53	3.64	91.54

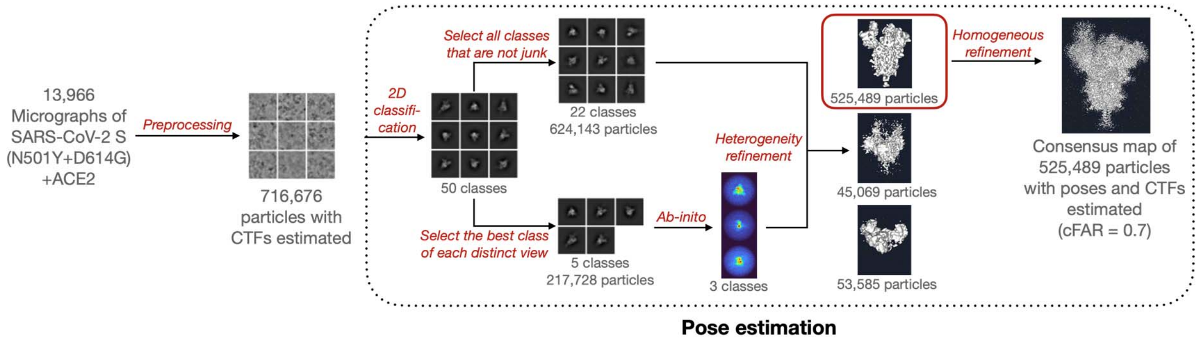

Figure S3 Workflow in CryoSPARC (Punjani *et al.*, 2017) to obtain clean particles with estimated contrast transfer functions (CTFs) and poses. 716,676 particles were extracted during preprocessing, from which 2D classification identified the best classes of each distinct view. Three ab-initio models were constructed from the particles of those best classes. After that, heterogeneous refinement with three classes was performed with the ab-initio models and the particles from the classes that were not identified as junk to produce three volumes. The final density map, consisting of 525,489 particles with estimated CTFs and poses, was constructed by homogeneous refinement on the particles from the only volume corresponding to an intact SARS-CoV-2 spike structure from heterogeneous refinement. Pose diagnostics yielded a Conical FSC Area Ratio (cFAR) score of 0.7.

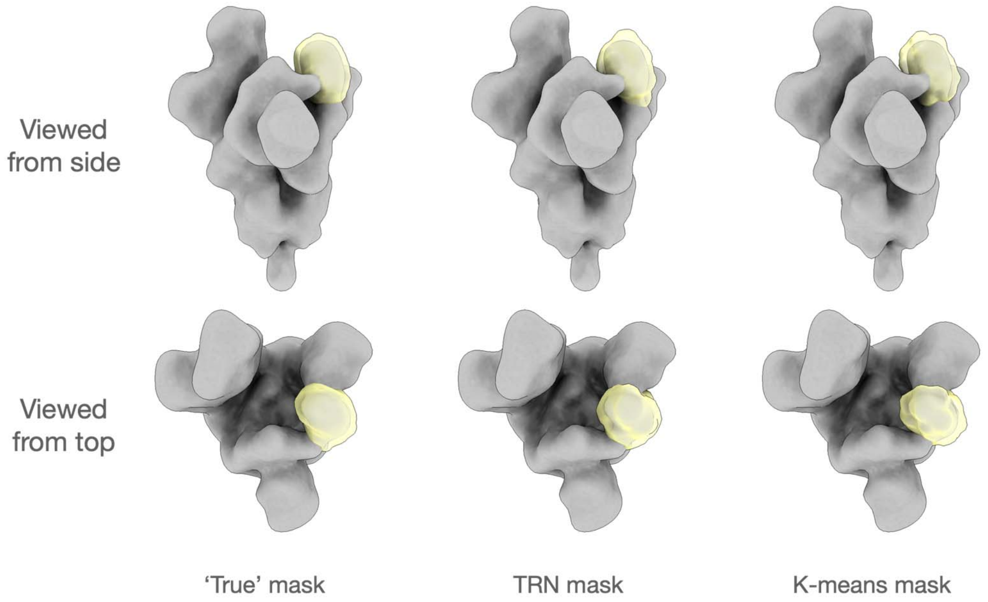

Figure S4 A comparison of the three masks generated manually, by topology representing network (TRN) and by k-means shows comparable performance between TRN and k-means in the mask generation task. A mask created manually on one of the RBDs is present as the ideal mask we aim for. The automated masking algorithm with TRN and k-means was applied to generate another two masks. Masks generated with TRN and k-means captured the desired region with similar accuracy.

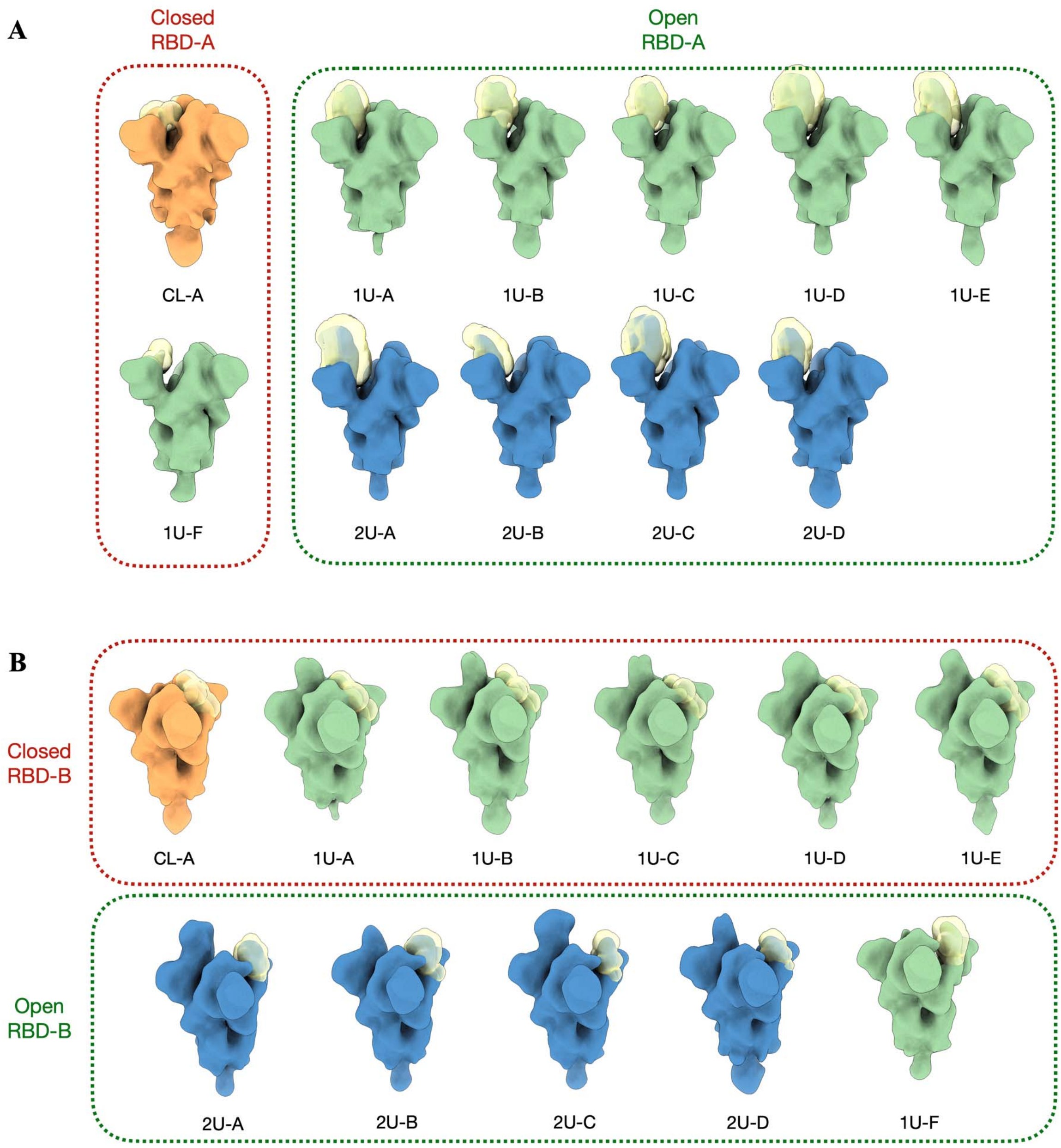

Figure S5 Main volume masks generated for (A) RBD-A and (B) RBD-B through the automated masking algorithm.

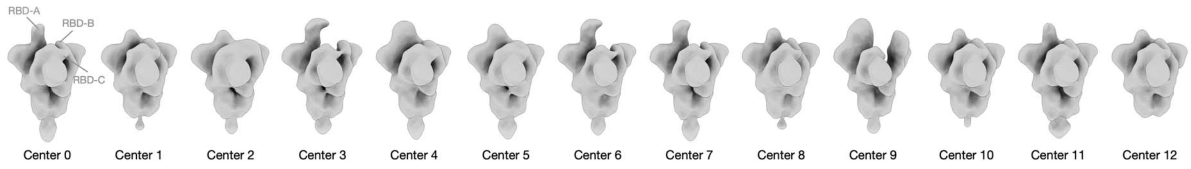

Figure S6 Thirteen main volumes (determined by the elbow rule) were found by k-means from the embeddings given by cryoDRGN.

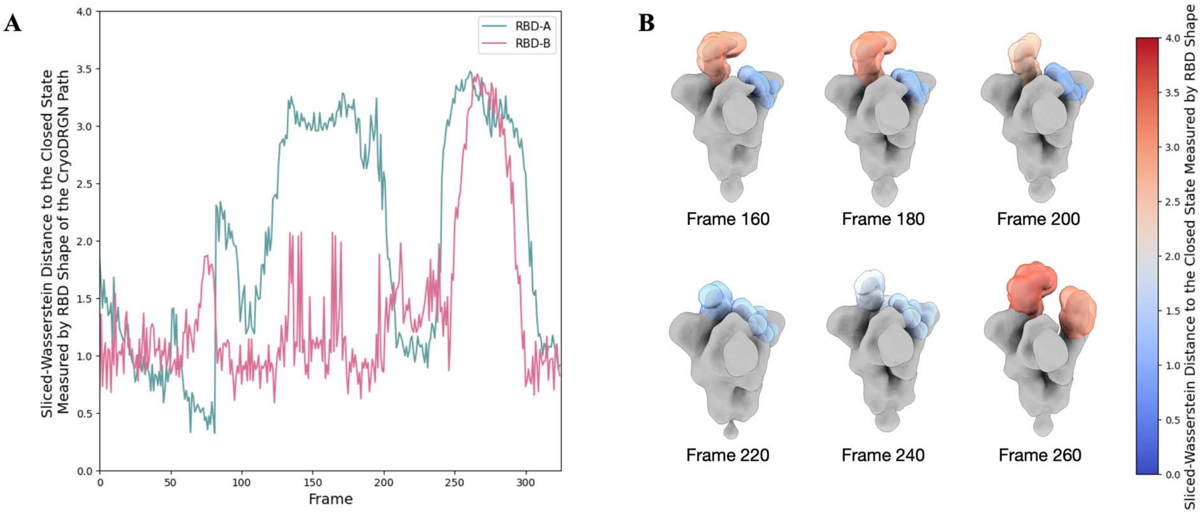

Figure S7 Cooperativity between RBD-A and RBD-B is also present in the trajectory traversing the latent space by cryoDRGN. (A) Changes in the sliced-Wasserstein distance (SWD) with the closed state demonstrate that the only change in RBD-B, occurring at around frame 250, was accompanied by the decrease in RBD-A's SWD from frame 160 to 240, which then increased at the same time with the SWD in RBD-B. (B) Density maps with RBD-A and RBD-B masked show the cooperativity between RBD-A and RBD-B from frame 160 to frame 260.
